# Anterior Fusion and Long-Term Cervical Mobility in Patients With Traumatic Spinal Cord Injury: An Observational Study

**DOI:** 10.7759/cureus.45549

**Published:** 2023-09-19

**Authors:** Yannick Rau, Roland Thietje, Ludwig Matrisch, Sven Hirschfeld

**Affiliations:** 1 Arbeitsgruppe (AG) Forschung (Working Group Research), University of Lübeck, Lübeck, DEU; 2 Spinal Cord Injury Center, BG Klinikum Hamburg, Hamburg, DEU

**Keywords:** interbody fusion, cervical mobility, rehabilitation, spinal trauma, cervical spine, spinal cord injury (sci)

## Abstract

Objective

This study aims to determine and quantify the impairment of cervical mobility and range of motion in patients with traumatic spinal cord injury (SCI) and subsequent cervical subaxial fusion surgery.

Methods

A total of 89 patients who underwent interbody fusion of the cervical spine and were admitted to the Spinal Cord Injury Center of the BG Klinikum Hamburg, Germany between 2003 and 2018 were examined after their in-facility rehabilitation was successfully completed. Reclination, inclination, tilt, and rotation of the cervical spine were examined and documented in addition to overall patient characteristics and fusion extent.

Results

We could identify fusion length and age to be independently negatively correlated with the cervical range of motion in different degrees of movement. We could also show a significant decrease in cervical mobility within our patients when compared to healthy adults. The ability to tilt and rotate the cervical spine was particularly impaired.

Conclusions

Patients with traumatic SCI and intervertebral fusion suffer from significant impairment of mobility in different degrees of movement. This knowledge can be used to evaluate the rehabilitative challenges and reintegrative needs of individuals after traumatic SCI. Rehabilitation should be adjusted accordingly.

## Introduction

Current research from Germany estimates a yearly spinal cord injury (SCI) incidence of approximately 15.73 per million inhabitants. Around 58.7% of those injuries are reported to be related to the cervical spinal cord [1]. Even though SCI of the cervical spine is still rare, if it occurs, it often presents a life-changing event, especially when surgical intervention is required.

Surgical intervention for discoligamentous injuries usually consists of decompression and fusion of adjoining vertebrae to restore stability if necessary. Different approaches and techniques are possible [2]. Usually, modern fusion systems contain a combination of interbody cages and osteosynthesis. The approach and selection of fusion materials are mainly influenced by the surgeon’s assessment and experience. Surgery can be performed via an anterior, posterior, or combined approach. Recent publications describe the advantages of anterior fusions, but a definitive conclusion cannot be given yet [3,4]. However, within the last 20 years, anterior cervical interbody fusion (ACIF) and combined methods have become the gold standard in Germany and are therefore the most implanted in SCI patients. Regardless of the implemented method, all patients suffer from loss of mobility and range of motion of the cervical spine. This is detrimental, especially for tetraplegic individuals.

The degrees of mobility of the cervical spine presented in this study are inclination, reclination, tilt, and rotation. Inclination is defined as flexion, whereas reclination is defined as extension in the sagittal layer. Tilt is defined as lateral flexion in the frontal plane, and rotation is defined as rotation around the longitudinal axis. Cervical mobility and range of motion in healthy adult non-SCI patients were reported in other literature and can be used as a control [5,6].

The aim of this study is to evaluate how cervical fusion affects cervical range of motion. To achieve this goal, the range of motion in rehabilitated patients was examined and analyzed regarding possible correlations with the extent of surgery and age. The results were also correlated with healthy adult non-SCI patients from other publications.

## Materials and methods

**Figure 1:**
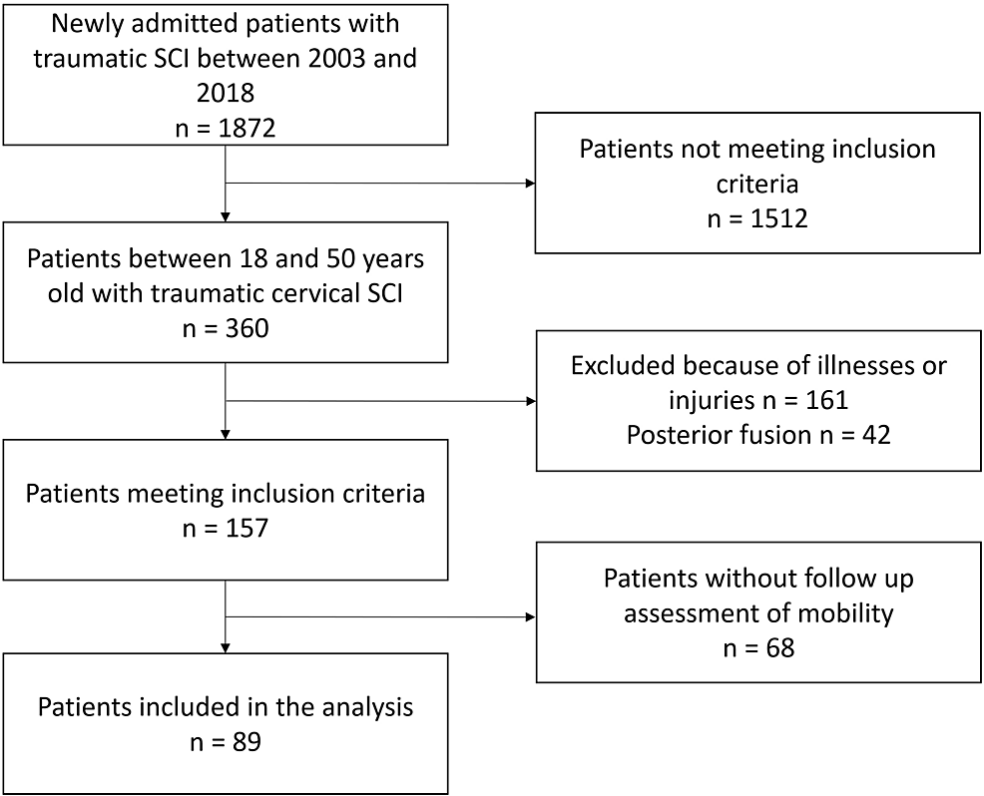
Patient enrollment SCI: Spinal cord injury, n: Number of patients

We included participants between the ages of 18 and 50 who suffered from traumatic cervical spine injuries and cervical spinal cord damage. Patients were treated via subaxial interbody fusion and initiated their rehabilitation at a level 1 trauma hospital in Hamburg from 2003 to 2018. An age of 50 years was the cutoff value, which is somewhat arbitrary but has been established in previous publications regarding rehabilitation success in individuals with SCI [7-10]. Patients with additional neurological or non-neurological impairments or consuming illnesses were excluded to further adjust for confounding factors. Figure 1 presents the patient selection process.

The range of motion was assessed by trained examiners via a well-established goniometer on patients seated in a resting posture. The assessment was performed approximately 12 months after rehabilitation was successfully completed. The level of injury and severity of impairment were determined by the American Spinal Injury Association (ASIA) impairment scale [11]. We also analyzed the functional outcome as presented by the Spinal Cord Independence Measure (II) (SCIM). We used the difference between SCIM values at the time of admission and the range of motion assessment. Respective SCIM values are only available for 83 out of the 89 included patients.

Statistical analysis

For this analysis, Jamovi version 2.3.11.0 (The Jamovi Project, Sydney, AU), an R-based tool, was used. The mean and standard deviation (SD) were supplied if possible. Frequencies or percentages were added if the values were categorical. The correlation was determined via Pearson’s r. Positive r-values were interpreted as positive correlations. Group comparison was performed via Welch’s t-test. To combine more than one independent variable, multiple linear regression was performed. For all analyses, an alpha error probability limit of 0.05 was established.

Ethical considerations

As data was pseudonymized at the time of extraction from the clinical database, the data could be processed without participants' explicit consent according to local law (§ 12 HmbKHG). Data was extracted and collected as part of a larger study, and the study protocol was presented to the Ethical Committee of the University of Lübeck, Germany, which granted permission for the study (approval no. 20-336).

## Results

A total of 89 patients were subject to analyses. Table 1 displays their epidemiological characteristics. The mean age was determined at 28.9 (SD 8.4) years old. Twenty-two patients underwent monosegmental fusion, 47 patients underwent bisegmental fusion, and 40 patients underwent fusion of three or more segments.

**Table 1:** Epidemiological characteristics of the study population The neurological levels of injuries, C3 to C7, were determined via the ASIA impairment scale at the time of admission. ASIA: American Spinal Injury Association, n: Number of patients

Attributes	Group	n	Relative
Gender	Male	79	88.8%
	Female	10	11.2%
Neurological level of injury	C3	18	20.2%
	C4	35	39.3%
	C5	26	29.2%
	C6	4	4.5%
	C7	6	6.7%
ASIA impairment scale type	A	42	47.2%
	B	9	10.1%
	C	21	23.6%
	D	17	19.1%
Cause	Motor vehicle accidents	27	30.3%
	Jumps in shallow waters	29	32.6%
	Fall inflicted injuries	16	18.0%
	Other	17	19.1%

Seventy-five patients were subjected to ACIF alone, while 14 were stabilized with the combined approach of ACIF and dorsal instrumentation. A t-test comparison between both groups showed only a significant difference (p = 0.007) between rotational ability and surgical approach, with the sole anterior approach offering a higher range of motion and a mean of 122.3° (SD 19.2°) versus 98.9° (SD 27.1°). When divided further into one-level and multi-level fusion, rotation only remained significantly greater in patients with sole ACI multi-level fusion. Combined or sole anterior cervical single-level fusions did not differ significantly. All other degrees of freedom differed non-significantly. No significant differences between the 10 women and 79 men could be found with regard to range of motion of any degree (p > 0.05).

Table 2 shows the overall range of motion in degrees within the population. Neither rotation nor tilt differed significantly between the left and right sides. However, a greater loss in mobility was noted (1.29°), although non-significant, when performing those movements on the right side in comparison to the left side.

**Table 2:** Overall range of motion in the collective Fusion length is represented as the number of fusion levels (e.g., 1 level = 2 fused vertebrae, 2 level = 3 fused vertebrae). n: Number of patients, SD: Standard deviation

Group	Inclination	Reclination	Rotation right	Rotation left	Tilt right	Tilt left
	Mean±SD	Mean±SD	Mean±SD	Mean±SD	Mean±SD	Mean±SD
All patients (n = 89)	37.36°±6.17°	54.89°±9.53°	58.65°±11.53°	59.94°±11.69°	34.05°±8.56°	34.27°±9.28°
Fusion length						
1 level (n = 22)	35.68°±7.76°	54.09°±8.95°	57.50°±12.51°	58.37°±13.47°	36.59°± 7.76°	35.68°±9.17°
2 levels (n = 47)	38.19°±3.52°	55.96°±8.05°	60.64°±9.53°	61.06°±9.38°	34.68°± 6.71°	35.53°±7.61°
3 levels or more (n = 20)	37.25°±8.66°	53.25°±13.01°	55.25°±14.09°	58.75°±14.59°	29.75°±1.64°	29.75°±1.75°

Table 3 shows a correlation matrix for all degrees of freedom and fusion length. All degrees correlated positively with each other, meaning a higher ability to rotate was associated with a higher ability to tilt, etc. Reclination, rotation, and tilt also correlated negatively with fusion length. Longer fusion was associated with reduced range of motion in these three degrees, while inclination was not affected.

**Table 3:** Correlation matrix of degrees of freedom and length of fusion

Degree of freedom	Variable	Inclination	Reclination	Rotation	Tilt	Fusion length
Inclination	Pearson's r	—				
	p-value	—				
Reclination	Pearson's r	0.711	—			
	p-value	< 0.001	—			
Rotation	Pearson's r	0.565	0.652	—		
	p-value	<0 .001	< 0.001	—		
Tilt	Pearson's r	0.597	0.616	0.512	—	
	p-value	< 0.001	< 0.001	< 0.001	—	
Fusion length	Pearson's r	-0.079	-0.215	-0.215	-0.365	—
	p-value	0.461	0.042	0.042	< 0.001	—

Table 4 shows the results if rotation is further divided into right and left rotations. Interestingly, while left rotation does not significantly correlate with fusion length, rotation to the right does negatively correlate with increased fusion length. The same could not be reproduced with tilt degree to the right or left, as shown in Table 5.

**Table 4:** Correlation matrix of rotation to the right and left and fusion length

Degree of freedom	Variable	Rotation right	Rotation left	Fusion length
Rotation right	Pearson's r	—		
	p-value	—		
Rotation left	Pearson's r	0.818	—	
	p-value	< 0.001	—	
Fusion length	Pearson's r	-0.245	-0.152	—
	p-value	0.020	0.154	—

**Table 5:** Correlation matrix of right and left tilt and fusion length

Degree of freedom	Variable	Tilt right	Tilt left	Fusion length
Tilt right	Pearson's r	—		
	p-value	—		
Tilt left	Pearson's r	0.821	—	
	p-value	< 0.001	—	
Fusion length	Pearson's r	-0.386	-0.320	—
	p-value	< 0.001	0.002	—

Table 6 shows an additional analysis of the degree of freedom and its correlation with age as previous publications have found a negative correlation between age and cervical mobility [12,13].

**Table 6:** Correlation matrix of the degree of freedom and age

Degree of freedom	Variable	Rotation	Inclination	Reclination	Tilt	Age
Rotation	Pearson's r	—				
	p-value	—				
Inclination	Pearson's r	0.407	—			
	p-value	< 0.001	—			
Reclination	Pearson's r	0.532	0.598	—		
	p-value	< 0.001	< 0.001	—		
Tilt	Pearson's r	0.399	0.498	0.524	—	
	p-value	< 0.001	< 0.001	< 0.001	—	
Age	Pearson's r	-0.301	-0.185	-0.315	-0.232	—
	p-value	0.004	0.083	0.003	0.028	—

**Figure 2:**
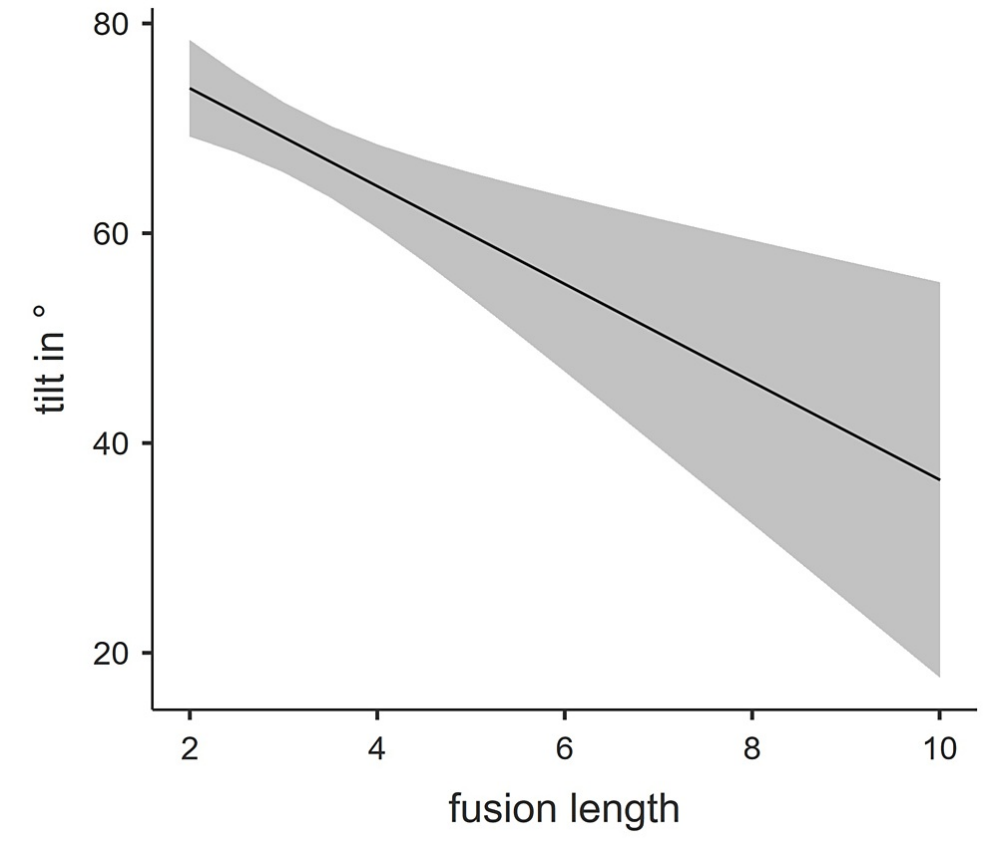
Estimated marginal means plot of tilt Fusion length is represented as the number of fused vertebrae.

**Figure 3:**
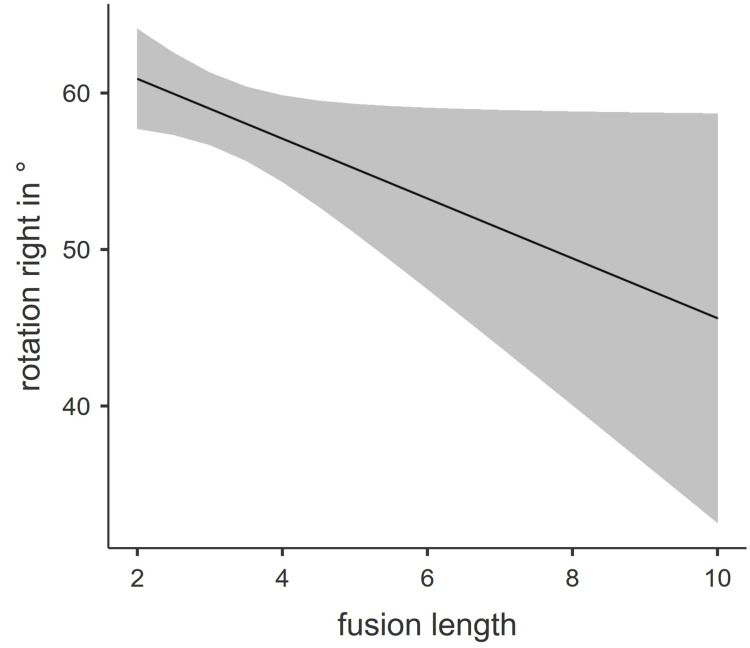
Estimated marginal means plot of rotation to the right Fusion length is represented as the number of fused vertebrae.

These findings could be replicated for rotation, reclination, and tilt. All three correlated significantly and negatively with age. Combining our findings into one multiple linear regression model, we found that rotation to the right (Nagelkerke’s R = 0.35) and tilt (Nagelkerke’s R = 0.41) both correlated significantly with fusion length adjusted for age. An estimated marginal means plot is provided for both analyses in Figures 2-3.

A correlation between cervical mobility and level of injury, as shown in Table 1, was not observed (all p > 0.05). A comparative analysis of our findings to the data from the studies conducted by Feng et al. and Fiebert et al. on cervical range of motion in 20 and 46 healthy adults, respectively, without fusion surgery or other predetermined illnesses, was performed to determine how severe the impairment of our patients presents itself [5,6]. The comparative analysis as shown in Table 7 resulted in a significant difference between our patients and the healthy population across varying degrees of freedom. Especially large differences could be observed between the groups in their abilities to rotate and incline the cervical spine.

**Table 7:** Comparison to healthy adults

Degree of freedom	Other studies	This study’s population	t-test p-value
Feng et al. findings [6] (n=20)			
Inclination	60.15°±8.46°	37.36°±6.17°	< 0.001
Reclination	64.75°±10.74°	54.89°±9.53°	< 0.001
Rotation left	82.1°±2.26°	59.94°±11.69°	< 0.001
Rotation right	82.65°±4.19°	58.65°±11.53°	< 0.001
Tilt left	54.4°±4.56°	34.27°±9.28°	< 0.001
Tilt right	49.98°±2.17°	34.05°±8.56°	< 0.001
Fiebert et al. findings [5] (n=46)			
Inclination	47.5°±14.1°	37.36°±6.17°	< 0.001
Reclination	69.5°±10.8°	54.89°±9.53°	< 0.001
Rotation left	67.2°±8.2°	59.94°±11.69°	< 0.001
Rotation right	65.1°±7.6°	58.65°±11.53°	0.0013
Tilt left	40.8°±7.0°	34.27°±9.28°	< 0.001
Tilt right	38.9°±7.0°	34.05°±8.56°	< 0.001

An individualized approach with subgroups divided by fusion length, as shown in Table 2, did not show different results for short fusion lengths such as monosegmental fusion, except for the mean differences increasing with additional fused segments. To quantify the impact of the observed movement impairment on the functional results of the patients, we also correlated cervical rotation, tilt, inclination, and reclination with the spinal cord independence measure (SCIM) II differences between the time of admission and range of motion assessment (Table 8). It could be shown that only the extension of the rotational ability of the cervical spine correlated positively with the functional outcome as presented by the SCIM difference.

**Table 8:** Correlation between the degrees of freedom and SCIM (II) differences between the time of admission and assessment T1: Date of admission, T2: Date of range of motion assessment, SCIM: Spinal cord independence measure

Degree of freedom	Variable	SCIM T2/T1 difference	Rotation	Tilt	Inclination	Reclination
SCIM T2/T1 difference	Pearson's r	—				
	p-value	—				
Rotation	Pearson's r	0.259	—			
	p-value	0.018	—			
Tilt	Pearson's r	0.008	0.399	—		
	p-value	0.945	< 0.001	—		
Inclination	Pearson's r	-0.001	0.407	0.498	—	
	p-value	0.994	< 0.001	< 0.001	—	
Reclination	Pearson's r	0.062	0.532	0.524	0.598	—
	p-value	0.575	< 0.001	< 0.001	< 0.001	—

## Discussion

Results

We conducted a monocentric cohort study on 89 patients with cervical SCI and anterior fusion. We analyzed their cervical mobility and assessed potential influences such as age and fusion length. We also compared our population to healthy adults in the literature. Our findings show that patients with SCI undergoing cervical interbody fusion are significantly limited in their ability to rotate, tilt, incline, and recline their cervical spine when compared to healthy adults without SCI. This is in line with findings on cervical fusion in patients without SCI [14].

We could also show a significant correlation between fusion length, reclination, and tilt, as well as rotation to the right. While a non-significant difference between right and left rotation has been previously reported in healthy adults and is also present here, this particular phenomenon has not yet been discovered [5,6]. A possible explanation could be the preferred surgical approach in ACIF. Anterior cervical interbody fusion is primarily performed through an incision on the right-hand side of the patient’s neck. The preferred procedure is the Smith-Robinson procedure or a modified version of it [15-18]. This approach may, however, result in unilateral scarring and heterotopic ossification, as shown by other authors investigating cervical disc replacement [19,20]. The amount of heterotropic ossification around the disc replacement sites correlates with fusion length, according to one study by Wu et al. [19]. Disc replacement cannot be directly compared to fusion surgery as present in our study, but it may give a hint as to what may cause the observed differences in movement. The current literature does not account for the surgical approach or whether, as a consequence, range of motion may be impaired unilaterally when it comes to rotation. This should be further investigated by also controlling for the incision side in future studies analyzing heterotropic ossification.

Our findings regarding the correlation between fusion length and reclination are in line with a previous study by Limanówka and Sagan, on a small number of patients who underwent ACIF in degenerative diseases that reported a correlation between fusion length and range of motion in the sagittal plane [21]. A general reduction in reclination and rotational ability compared to healthy adults after single-level anterior fusion in patients with degenerative disc disease was also reported by Ylinen et al. and could also be supported by our findings [22].

An additional significant difference in range of motion in rotation could be observed between anterior and combined fusion patients. A systematic review published by Lu et al. suggested higher mobility in combined or hybrid approaches versus sole ACIF in multi-level fusions. This could not be replicated in this study. Instead, rotational ability that was not independently assessed by Lu et al. was determined to be significantly higher in multi-level ACIF patients as opposed to combined approaches [23]. This discrepancy may be explained by the fact that our collective only consisted of traumatic injury patients instead of degenerative spine surgery patients, and that in general, the combined approach in multi-level surgery is used for more severe and impairing injuries.

Age could also be determined to correlate significantly with range of motion. This has been previously shown by other authors in healthy adult non-SCI individuals as well as in orthopedic patients and was therefore expected to also affect our study population, even though we excluded patients over the age of 50 for this reason, and impairment by age until then is reported as low [12,13]. Other age groups could theoretically show other characteristics than our patient collective.

We could not observe significant correlations between injury level and range of motion. This was expected as muscular cervical mobility relies heavily on cranial nerve XI and, in some minor capacities, the cervical spinal nerves, with an emphasis on the upper segments [24]. Our population did not exceed paralysis levels of C3.

It has been previously shown that fusion length negatively correlates with functional impairment after rehabilitation in patients with cervical SCI and spinal fusion [25]. In this study, we also correlated the range of motion of the cervical spine with the functional impairment and found that the impairment of rotation in particular is positively correlated with the functional outcome. This can be used to improve rehabilitation by the respective professionals. Occupational therapy and physical therapy in particular may be able to adapt their therapeutic strategies, goals, and aids accordingly. While the impairment of movement may not be positively influenced as the cervical fusion restricts movement indefinitely, strategies to negate the negative impact of rotational impairment may be employed (e.g., wheelchairs with customized control mechanisms).

Strengths and limitations

The large sample size is one of the main strengths of this study. Similar research usually relies on smaller groups and subsequently smaller subgroups with less ability to generalize results [6,14,26]. The overall epidemiological characteristics of our population, such as age distribution within the limits of 18 to 50 years old, gender distribution, and incomplete versus complete tetraplegia, are in line with other studies of cervical spinal cord injury [27,28]. Additionally, our exclusion criteria reduce the impact of comorbidities and injuries on the results and confound the impact of fusion surgery.

The monocentric design of the study may create institutional bias. The fact that rehabilitation processes are highly individual also means that the time from surgery until assessment may differ by months between patients. The age limits at enrollment help to adjust for confounding factors but also reduce generalization to other age groups. The utilization of goniometers has inherent reliability problems and may result in inaccuracies in measurement [29]. However, the large cohort somewhat negates these issues. Overall, the study design provides a solid basis to assess long-term cervical mobility in patients with SCI and cervical fusion.

## Conclusions

Spinal cord injury patients are often severely impaired. The impairment is dependent on a variety of factors, but in general, it is influenced by the level of injury and neurological severity. Both can be reliably determined via the ASIA impairment scale and magnetic resonance imaging. Patients’ functional outcomes, however, depend on more than just neurological outcomes. Especially those suffering from severe cervical SCIs are heavily reliant on cervical mobility in daily tasks that involve tool use in general (e.g., personal hygiene or mobility). The knowledge about reduction in cervical range of motion and the relation between rotational impairment and functional outcome after fusion surgery, especially considering its correlation with fusion length, helps professionals better determine the extent of impairment and may be beneficial in adjusting therapeutic strategies for occupational and physical therapy.
